# Distinct Molecular Features of Different Macroscopic Subtypes of Colorectal Neoplasms

**DOI:** 10.1371/journal.pone.0103822

**Published:** 2014-08-05

**Authors:** Kenichi Konda, Kazuo Konishi, Toshiko Yamochi, Yoichi M. Ito, Hisako Nozawa, Masayuki Tojo, Kensuke Shinmura, Mari Kogo, Atsushi Katagiri, Yutaro Kubota, Takashi Muramoto, Yuichiro Yano, Yoshiya Kobayashi, Toshihiro Kihara, Teppei Tagawa, Reiko Makino, Masafumi Takimoto, Michio Imawari, Hitoshi Yoshida

**Affiliations:** 1 Division of Gastroenterology, Department of Medicine, Showa University School of Medicine, Tokyo, Japan; 2 Department of Pathology, Showa University School of Medicine, Tokyo, Japan; 3 Clinical Collaborating laboratory, Showa University School of Medicine, Tokyo, Japan; 4 Department of Hospital Pharmaceutics, Showa University School of Pharmacy, Tokyo, Japan; 5 Department of Biostatistics, Hokkaido University Graduate School of Medicine, Sapporo, Japan; Duke-NUS, Singapore

## Abstract

**Background:**

Colorectal adenoma develops into cancer with the accumulation of genetic and epigenetic changes. We studied the underlying molecular and clinicopathological features to better understand the heterogeneity of colorectal neoplasms (CRNs).

**Methods:**

We evaluated both genetic (mutations of *KRAS*, *BRAF*, *TP53*, and *PIK3CA*, and microsatellite instability [MSI]) and epigenetic (methylation status of nine genes or sequences, including the CpG island methylator phenotype [CIMP] markers) alterations in 158 CRNs including 56 polypoid neoplasms (PNs), 25 granular type laterally spreading tumors (LST-Gs), 48 non-granular type LSTs (LST-NGs), 19 depressed neoplasms (DNs) and 10 small flat-elevated neoplasms (S-FNs) on the basis of macroscopic appearance.

**Results:**

S-FNs showed few molecular changes except *SFRP1* methylation. Significant differences in the frequency of *KRAS* mutations were observed among subtypes (68% for LST-Gs, 36% for PNs, 16% for DNs and 6% for LST-NGs) (P<0.001). By contrast, the frequency of *TP53* mutation was higher in DNs than PNs or LST-Gs (32% vs. 5% or 0%, respectively) (P<0.007). We also observed significant differences in the frequency of CIMP between LST-Gs and LST-NGs or PNs (32% vs. 6% or 5%, respectively) (P<0.005). Moreover, the methylation level of LINE-1 was significantly lower in DNs or LST-Gs than in PNs (58.3% or 60.5% vs. 63.2%, P<0.05). *PIK3CA* mutations were detected only in LSTs. Finally, multivariate analyses showed that macroscopic morphologies were significantly associated with an increased risk of molecular changes (PN or LST-G for *KRAS* mutation, odds ratio [OR] 9.11; LST-NG or DN for *TP53* mutation, OR 5.30; LST-G for *PIK3CA* mutation, OR 26.53; LST-G or DN for LINE-1 hypomethylation, OR 3.41).

**Conclusion:**

We demonstrated that CRNs could be classified into five macroscopic subtypes according to clinicopathological and molecular differences, suggesting that different mechanisms are involved in the pathogenesis of colorectal tumorigenesis.

## Introduction

Colorectal cancer (CRC) can develop via various molecular pathways. Most CRCs develop over a long period of time through a multistep process called the adenoma-carcinoma sequence [1]. Approximately two-thirds of sporadic CRCs arise from conventional adenomas and usually show a protruding (polypoid) macroscopic appearance. The process of colorectal carcinogenesis often begins with the inactivation of the *APC*/β-catenin signaling pathway (the Vogelstein model), followed by *KRAS* and *TP53* mutations [2]. However, serrated adenomas (SAs), particularly sessile serrated adenoma/polyps (SSA/Ps), have been described as the immediate precursors for CRCs that develop via an alternative pathway consisting of the CpG island methylator phenotype (CIMP) and *BRAF* mutations [3], [4]. In addition, CIMP cancers may develop either via a mutator (microsatellite instability; MSI) pathway, or via a pathway that leads to microsatellite stability (MSS) [5]. However, additional pathways that are not fully understood may also contribute to colorectal carcinogenesis.

Recent studies [6]–[8] revealed that conventional (non-serrated) adenomas could be morphologically classified into polypoid neoplasms (PNs) and nonpolypoid neoplasms (NPNs; also referred to as flat and depressed neoplasms). PNs develop via the traditional adenoma-carcinoma sequence, and their tumorigenesis is characterized by loss of heterozygosity, which leads to the inactivation of tumor suppressor genes such as *APC* and *TP53* [2]. The mutation of *KRAS*, which activates the mitogen-activated protein kinase (MAPK) cascade and promotes malignant transformation, is a key event in the adenoma-carcinoma sequence [9]. In contrast, NPNs have a low frequency of *KRAS* mutation, and the chromosomal changes that occur during the development of NPNs are markedly different from those that occur during the progression of PNs [10]–[13]. We previously showed that NPNs have a higher frequency of MSI (MSI-H), an increased abnormal accumulation of phosphorylated MAPK protein, and a lower frequency of *KRAS* mutations than PNs [14]. In addition, An *et al.* [15] reported specific patterns of aberrant DNA methylation in CIMP-negative CRCs, particularly a decrease in global DNA methylation, and an increase in the age-related methylation of multiple genes such as *MGMT*, *RASSF1* and *SFRP1*.

Clinically, depressed neoplasms (DNs) are characterized by increased risk of malignancy as compared with PNs, even if they are small [16]. In addition, flat elevated neoplasms can be classified into small-flat adenoma and laterally spreading tumors (LSTs), which were initially reported by Kudo *et al.* [7], and are characterized by lateral extension along the luminal wall with a low vertical axis. These tumors are sub-categorized into granular type LST (LST-G) and non-granular type LST (LST-NG), based on different molecular features [17], [18]. NPNs are not easily detected during a colonoscopy and it is challenging for the colonoscopists to distinguish them from the normal mucosa [7]. Inadequate recognition of NPNs could result in the development of interval cancers. Molecular analysis of precursor lesions and early stage CRC should therefore be performed to gain a better understanding of the different pathways of CRC development. In addition, such analyses may be used for the implementation of the appropriate screening and therapeutic intervention programs for CRCs.

We hypothesized that the epigenetic and genetic features of CRC may be shared with macroscopic subtypes of conventional neoplasms such as adenomas and T1 carcinoma. In the present study, we tested this hypothesis by determining the genetic and epigenetic profiles of colorectal neoplasms (CRNs) by using molecular markers associated with the mechanism of colorectal carcinogenesis.

## Materials and Methods

### Patients and samples

A total of 158 CRNs from 153 patients who underwent endoscopic (n = 125) or surgical resection (n = 33) at Showa University Hospital were examined. The samples were selected solely based on tissue availability. Patients with inflammatory bowel disease or with a familial predisposition to cancers such as familial adenomatous polyposis or hereditary nonpolyposis colorectal cancer were excluded. The ethics committee of the Showa University School of Medicine approved the procedures for tissue collection and analysis, and written informed consent was obtained from each patient.

### Endoscopic evaluation and macroscopic classification

All patients were prepared for the procedure by administration of 1.8 L oral electrolyte lavage solution. Colonoscopists with extensive experience performed all examinations by using high-resolution video colonoscopes (CF-240ZI, CF-260AI, or CF-260HZI; Olympus Optical Co., Tokyo, Japan). CRNs were then prospectively classified as PN (Figure S1a) or NPN (Figure S1b-e) based on the Paris endoscopic classification [19]. Briefly, NPNs were defined as neoplasms showing slight mucosal elevation with a flat or slightly rounded surface and a height of less than half the diameter of the lesion. Histological examination indicated that NPNs typically displayed dysplastic mucosal thickness less than twice that of the adjacent nondysplastic mucosa. NPNs comprised flat-elevated (Figure S1b, d, e) and DNs (Figure S1c), depending on the presence of a depressed component. The flat-elevated lesions were subclassified into small flat-elevated neoplasms (S-FNs) (Figure S1b), LST-G (Figure S1d) and LST-NG (Figure S1e). Briefly, LSTs are defined by a large lateral diameter (>10 mm), a low vertical axis, and lateral extension along the luminal wall [20]. LST-Gs are composed of superficial spreading aggregates of nodules that form flat, broad-based lesions with a granulonodular and uneven surface, whereas LST-NGs have a flat smooth surface without granulonodular formation. In contrast, PNs presented with sessile, pedunculated or semipedunculated morphology (Figure S1a). The details of this macroscopic classification based on colonoscopic findings are summarized in Table S1 in File S1.

### Tissue samples and histological evaluation

Serial sections (3 µm) were obtained from paraffin-embedded blocks and prepared for hematoxylin and eosin (H&E) staining. All H&E-stained slides were analyzed by a senior pathologist (T.Y.) who was blinded to the endoscopic findings. Hyperplastic polyps and SAs were not included in this analysis.

To extract genomic DNA, 15 formalin-fixed paraffin-embedded samples and 143 frozen tissue samples were used. The frozen tissue samples were obtained from colonoscopic biopsy specimens and stored at -80°C. We distinguished between neoplastic and non-neoplastic areas of the specimens based on pit patterns observed during chromoendoscopic examination [21], [22]. DNA was extracted from frozen tissue samples by using standard proteinase K/phenol/chloroform methods.

Serial slides were obtained from the archival formalin-fixed, paraffin-embedded tumor tissues. One slide was stained with H&E for microdissection. After microdissection, DNA was extracted using the QIAamp DNA mini kit (QIAGEN Inc., Valencia, CA).

### Bisulfite polymerase chain reaction and pyrosequencing analysis of DNA methylation

Bisulfite treatment was performed as previously described [23], and 2–3 µL bisulfite-treated DNA was used as the template for polymerase chain reaction (PCR). The primers and PCR conditions used for the amplification of target genes have been described previously. The protocol for pyrosequencing, a quantitative tool for determining methylation density, has been described in detail [24]. Pyrosequencing can be used to measure the level of methylation at several CpG sites in a given promoter, and the methylation status of different sites is usually consistent. For each gene, the methylation percentage of all CpGs measured was averaged.

### Methylation-related genes and definition of CIMP

We studied 9 genes or sequences (MINT1, MINT2, MINT31, *CDKN2A*, *MLH1*, *MGMT*, *RASSF1*, *SFRP1*, and LINE-1) in this analysis. The PCR and sequencing primer sequences used were previously reported [24]–[26]. Sporadic CRCs can be classified into 2 groups, CIMP-positive and CIMP-negative according to the frequency of methylation of the CpG islands in the promoter of 5 genes (MINT1, MINT2, MINT31, *CDKN2A*, and *MLH1*) [27]. As determination of the CIMP status requires a quantitative tool, positive methylation status was defined as a methylation density greater than 15% [24]. A tumor was considered to be CIMP-positive if 2 or more CIMP markers were methylated, as described previously [27]. All other tumors were defined as CIMP-negative.

### *KRAS*, *BRAF*, *TP53* and *PIK3CA* gene mutations and MSI

Samples were analyzed using PCR-based pyrosequencing to determine the presence of activating mutations in codons 12 and 13 of *KRAS*, codon 600 of *BRAF*, and in exons 9 and 20 of *PIK3CA* [28]–[30]. Exons 5 to 8 of *TP53* and MSI were assessed following previously described protocols [31].

### Statistical analysis

Pyrosequencing provides a methylation level (%), which was analyzed as a continuous variable for the comparison of each gene with clinicopathological variables. The mean, median, ranges, and 95% confidence intervals (CI) were calculated.

Differences in the continuous variables (age, tumor size, and methylation density) among groups were analyzed by using the Kruskal-Wallis test. Post-hoc tests such as the Steel-Dwass method were used to compare differences in the continuous variables between groups, and P<0.05 was considered to be statistically significant. Categorical variables were compared between subtypes by using χ^2^ or Fisher's exact test when testing small samples. All tests were two-sided. P values were considered to be significant at a Bonferroni-corrected alpha of 0.05.

Logistic regression analysis using the stepwise method was performed to evaluate the relationship between molecular alterations of CRNs and gender, age, tumor location (proximal vs. distal), tumor size, macroscopic types, and carcinoma component (T1 cancer). In this analysis, gender, tumor location, macroscopic type, histology, and genetic alterations were considered as categorical variables, whereas age and tumor size were used as continuous variables. The odds ratio (OR) and 95% CI were determined for a variety of factors. P<0.05 was considered to be significant. All statistical analyses were performed with SPSS version 14.0 (SPSS, Inc., Tokyo, Japan) and JMP version 10 (SAS Institute, Inc., Cary, NC).

## Results

We analyzed the molecular features of 158 CRNs. Table 1 summarizes the patients' clinicopathological characteristics. No significant differences in the clinicopathological features were observed between all CRNs and those with high-grade dysplasia (HGD)/submucosal cancer (T1 cancer).

**Table 1:** Clinicopathological features of colorectal neoplasms.

				All tumors	HGD/T1 cancer
				*N* = 158	*N* = 77
Gender		Male		96 (63%)	45 (58%)
		Female		57 (37%)	32 (42%)
Age		(yrs)		68.1	68.2
		(range)		(37–89)	(43–89)
Tumor location		Proximal		75 (47%)	35 (45%)
		Distal		83 (53%)	42 (55%)
Size		(mm)		18.9	22.4
		(range)		(3–73)	(5–50)
Histology		Adenoma		118	NA
		LGD		81 (51%)	
		HGD		37 (23%)	
		T1 cancer*		40 (25%)	
Macroscopic type		PN		56 (35%)	26 (34%)
		LST-G		25 (16%)	9 (12%)
		LST-NG		48 (30%)	21 (27%)
		S-FN		10 (6%)	2 (3%)
		DN		19 (12%)	19 (25%)

*, all cases were submucosal cancers. Proximal, cecum, ascending and transverse colon; distal, descending and sigmoid colon, and rectum; LGD, low grade dysplasia; HGD, high grade dysplasia; PN, polypoid neoplasm; LST-G, granular type laterally spreading tumor; LST-NG, non-granular type LST; S-FN, small flat-elevated neoplasm; DN, depressed neoplasm; NA, not applicable.

### Clinicopatological and molecular features of macroscopic subtypes of colorectal neoplasms

Ten out of 158 CRN lesions were S-FNs, and had few molecular changes except *SFRP1* methylation (Table S2 in File S1). Therefore, four subtypes were further analyzed. The clinicopatological features of the four phenotypes of CRNs are summarized in Table 2. LST-Gs were significantly larger than the other subtypes (P<0.05 by Steel-Dwass). Regarding histological grade, we observed a significant difference between DNs and the other subtypes (P<0.0001).

**Table 2:** Clinicopathological and molecular characteristics among different macroscopic subtypes of CRNs.

		PN (%)	LST-G (%)	LST-NG (%)	DN (%)	*P* value*
		(N = 56)	(N = 25)	(N = 48)	(N = 19)	
Gender	Male	35 (63)	12 (48)	33 (69)	12 (63)	0.3873
	Female	21 (37)	13 (52)	15 (31)	7 (37)	
Age	mean, yrs	67.2	71.2	68.4	68.4	0.5900
	(range, yrs)	(40–88)	(55–89)	(53–82)	(43–87)	
Location	Proximal	22 (39)	13 (52)	25 (52)	9 (47)	0.5557
	Distal	34 (61)	12 (48)	23 (48)	10 (53)	
Size	mean, mm	16.9	29.0	18.8	18.5	0.0004
	(range, mm)	(4–40)	(12–73)	(10–39)	(7–35)	
Histology	LGD	30 (54)	16 (64)	27 (56)	0	<0.0001
	HGD + T1 cancer	26 (46)	9 (36)	21 (44)	19 (100)	
Frequency of gene alteration/phenotype						
KRAS	Mut +	20 (36)	17 (68)	3 (6)	3 (16)	<0.0001
	Mut −	36 (64)	8 (32)	45 (94)	16 (84)	
BRAF	Mut +	1 (2)	0	0	2 (11)	0.0378
	Mut −	55 (98)	25 (100)	48 (100)	17 (89)	
TP53	Mut +	3 (5)	0	6 (13)	6 (32)	0.0028
	Mut −	53 (95)	25 (100)	42 (87)	13 (68)	
PIK3CA	Mut +	0	4 (17)	1 (2)	0	0.0012
	Mut −	56 (100)	19 (83)	44 (98)	17 (100)	
MSI-H	presence	0	0	3 (6)	2 (11)	0.0713
	absence	56 (100)	25 (100)	45 (94)	17 (89)	
CIMP	presence	3 (5)	8 (32)	3 (6)	3 (16)	0.0028
	absence	53 (95)	17 (68)	45 (94)	16 (84)	
DNA methylation density (%)						
MGMT	Mean	13.0	8.5	8.7	8.2	0.0824
	95% CI	9.0–17.1	3.8–13.3	5.0–12.4	0.9–15.5	
SFRP1	Mean	49.5	59.7	39.5	39.7	<0.0001
	95% CI	45.2–53.8	54.7–64.7	35.4–43.7	34.8–44.6	
RASSF1	Mean	7.4	6.4	5.0	5.4	0.2562
	95% CI	5.1–9.7	3.5–9.4	2.9–7.1	1.5–9.3	
LINE-1	Mean	63.2	60.5	61.4	58.3	0.0002
	95% CI	62.0–64.4	59.0–62.1	60.2–62.6	55.7–60.9	

*, P values were calculated by Chi-square test or Kruskal-Wallis test. PN, polypoid neoplasm; LST-G, granular type laterally spreading tumor; LST-NG, non-granular type LST; DN, depressed neoplasm; proximal, cecum, ascending and transverse colon; distal, descending and sigmoid colon, and rectum; LGD, low grade dysplasia; HGD, high grade dysplasia; MSI-H, high frequency microsatellite instability; CIMP, CpG island methylator phenotype; Mut+, presence of mutation; Mut-, absence of mutation.

The molecular features of four subtypes of CRNs are shown in Table 2. Significant differences in the frequency of *KRAS*, *TP53*, *PIK3CA* mutations and CIMP were observed among the four subtypes. We found significant differences in the frequency of *KRAS* mutations among the subtypes (PNs vs. LST-NGs, P = 0.0003; LST-Gs vs. LST-NGs, P<0.0001; LST-Gs vs. DNs, P = 0.0008; PNs vs. LST-Gs, P = 0.0070, respectively). By contrast, the frequency of *TP53* mutation was higher in DNs than PNs or LST-Gs (DNs vs. PNs, P = 0.0066; DNs vs. LST-Gs, P = 0.0038, respectively). Significant differences in the frequency of CIMP were observed between LST-Gs and LST-NGs or PNs (LST-Gs vs. LST-NGs, P = 0.0035; LST-Gs vs. PNs, P = 0.0012, respectively). Although *MGMT* and *RASSF1* methylation density did not differ significantly between any subgroups, there were differences in the DNA methylation levels of *SFRP1* between two subtypes (P<0.05 by Steel-Dwass except LST-NGs vs. DNs). The DNA methylation level of LINE-1 was significantly lower in LST-Gs or DNs than in PNs (P<0.05 by Steel-Dwass).

### *PIK3CA* mutations in CRNs

Of 158 CRN samples, 151 were examined for *PIK3CA* mutation status. The frequency of *PIK3CA* mutation was uncommonly low in our studied samples (3%, 5/151; Table 2). However, the frequency was higher in LST-G tumors compared with the other sub-groups (LST-Gs, 17% [4/23]; LST-NGs, 2% [1/45]; PNs, 0% [0/56]; DNs, 0% [0/17]). Of these, the difference in the frequency of *PIK3CA* mutations between LST-Gs and PNs was statistically significant (P = 0.0076). Four of the five tumors with a *PIK3CA* mutation were diagnosed as HGD or T1 cancer. In addition, *PIK3CA* mutation was not significantly correlated with other molecular changes such as *KRAS* mutation or CIMP.

### Multivariate analysis

Finally, multivariate analysis was performed to determine whether molecular alterations can be predicted by clinicopathological variables (Tables 3 and Table S3 in File S1). In the multivariate analysis, PN/LST-G morphology and tumor size were significant risk factors for *KRAS* mutation, whereas LST-NG/DN morphology was the only significant risk factor for *TP53* mutation. The morphology of LST-G was also the only significant risk factor for *PIK3CA* mutation. Regarding epigenetic alterations, patient age and tumor size were significant risk factors for CIMP. In the multivariate analysis for risk factors for LINE-1 hypomethylation, LST-G/DN morphology and carcinoma components were significant risk factors. In addition, we validated the results of multivariate analysis (Table S4 in File S1).

**Table 3:** Molecular alterations in relation to clinicopathological findings (multivariate analysis).

	Multivariate analysis		
Risk factor	Odds ratio	95% CI	*P* value
	*KRAS* mutation		
PN or LST-G	9.11	3.46–24.0	<0.001
Size (mm)	1.07	1.03–1.12	0.001
	*TP53* mutation		
LST-NG or DN	5.30	1.41–19.99	0.014
	*PIK3CA* mutation		
LST-G	26.53	2.81–250.11	0.004
	CIMP		
Age (yrs)	1.14	1.06–1.23	0.001
Size (mm)	1.12	1.05–1.19	0.001
	LINE-1 hypomethylation*		
LST-G or DN	3.41	1.54–7.58	0.003
Histology (T1 cancer)	4.40	1.93–10.04	<0.001

*, We used the median of methylation density of LST-G and DNs (59%) as a cut-off value for LINE-1 hypomethylation. CIMP, CpG island methylator phenotype; PN, polypoid neoplasm; LST-G, granular type laterally spreading tumor; LST-NG, non-granular type LST; DN, depressed neoplasm.

## Discussion

In the present study, we identified the distinct features of five macroscopic phenotypes of conventional neoplasms based on genetic alterations and DNA methylation profiles as follows: PN, high frequency of *KRAS* mutation; LST-G, high frequency of CIMP, *KRAS* and *PIK3CA* mutation and LINE-1 hypomethylation; LST-NG, relatively high frequency of *TP53* mutation; DN, high frequency of *TP53* mutation and LINE-1 hypomethylation; S-FN, rare methylation changes and genetic alterations. These five subtypes are correlated with the diversity of colorectal tumorigenesis. Our data also suggest that the five subtypes of CRNs differ clinically. For example, LST-G cases tended to be larger, whereas DN lesions more frequently had advanced neoplasms. However, S-FNs showed no significant molecular features in our analysis. Moreover, whether these macroscopic subtypes reflect tumorigenesis from distinct precursor cells, or represent distinct diseases that affect the same precursor cells via different environmental or epidemiological factors remains unknown. Nevertheless, our analyses reveal that they are sufficiently distinct to confirm the molecular heterogeneity of colorectal tumorigenesis and to merit consideration in clinical management.

Previous studies reported flat and depressed (refer to nonpolypoid) neoplasms are associated with high risk of malignant potential [8], [32]. However, in another study, no significant differences in the malignant potential between flat and polypoid adenomas were observed [33]. This discrepancy between studies could be attributed to differences in the macroscopic morphology of NPNs. In the present study, we showed the heterogeneity of molecular features among NPNs. Although the sample size was small, S-FNs showed few molecular changes associated with malignant progression, as reported previously by Morita *et al.* [34]. The remaining subtypes of NPNs had significant molecular signatures. Although a molecular analysis of NPNs was performed in several studies, different results were reported regarding molecular changes [17], [35]–[37]. Clinically, DNs rapidly progress to invasive cancers even when they are small [38]. LST-Gs with large nodules or LST-NGs with depression tend to show histological invasion into the submucosa [39]. However, S-FNs are often characterized by the presence of low-grade adenomas. Therefore, morphological differences between NPNs should be considered in the design of appropriate screening and therapeutic intervention programs. Whether S-FNs could progress to other subtypes of CRNs remains to be determined.

Recent studies have proposed a model for colorectal tumorigenesis that is consistent with three distinct molecular pathways (serrated, alternative, and traditional pathways) based on CIMP and MSI status, and *BRAF*/*KRAS* mutations [3], [26]. We showed that macroscopic appearance was significantly associated with the molecular phenotypes. Leggett and Whitehall [3] described an alternate pathway that is likely characterized by CIMP, *KRAS* mutation, and MSS, and they showed that no precursor lesion has been associated with this subgroup. However, the molecular features of this subgroup correspond to those of LST-G cases. On the other hands, DNs were characterized by LINE-1 hypomethylation and *TP53* mutation, but not *KRAS*/*BRAF* mutation. CRCs with these molecular features commonly have a poorer prognosis than other CRCs [40]. This may be associated with the aggressive behavior of DNs. This macroscopic classification is also clinically relevant, since the macroscopic appearance of CRNs could be used to predict their molecular features. This could lead to better understanding of the pathogenesis of CRCs and improve the management of premalignant lesions specific to each macroscopic subtype.

In the present study, we evaluated four genetic and nine epigenetic alterations. However, which molecular markers can identify the specific phenotypes of CRCs remains controversial. We selected markers to represent the distinct molecular features of CRCs. Alterations of these markers have been shown to be critical events during colorectal carcinogenesis [3]. However, few significant molecular abnormalities were observed in S-FN and LST-NG lesions using these molecular markers. Important challenges associated with the analysis of CRNs are heterogeneity between tumors, and poor reproducibility. Importantly, some studies may have been limited by bias caused by the small sample size; therefore, a large sample size is critical to examine the molecular features with adequate statistical power. In addition, possible disparities in the samples and sample collection methods among different studies, such as differences in the ethnicity of populations, and presence or absence of pretreatment may contribute to differing results. Advanced rectal cancer is treated with preoperative radiation in clinical practice, which could also cause bias and artifacts. Analysis of our study population shows that the sample size may be small and that there are different numbers of each macroscopic subtype. The small sample size could affect the estimate of the prevalence of molecular alterations. In addition, the smaller subgroups such as S-FNs and DNs could affect the power to detect specific molecular features between subgroups. Our findings should therefore be confirmed in additional sample sets.

Another limitation is that SAs were not included in this analysis. Many investigators have reported the relevance of SAs in colorectal oncogenesis [3]–[5]. Although both SAs, especially SSA/Ps, and non-SAs (conventional adenomas) frequently show flat-elevated morphology, they show different molecular features. Thus, we focused on the relationship between the macroscopic types and molecular features of conventional CRNs (non-serrated histology). However, we attempted to evaluate the clinicopathological and molecular features in SAs on the basis of our previously reported data (Table S5 in File S1) [41]. Although the sample size was small, the frequency of CIMP tended to be higher in LST type than other types of SAs. However, we found no significant differences in any molecular alterations such as *KRAS*/*BRAF* mutations, and MSI-H among macroscopic subtypes. Flat-elevated type SAs were more frequently located in the proximal colon and LST-NG type SAs often showed the histological finding of SSA/P.

Recent studies suggested that CRCs with *KRAS* mutations could be associated with a unique DNA methylation profile. CIMP2 CRCs exhibit increased age-related DNA methylation. Shen *et al*. [26] reported that the CIMP2 subgroup was highly correlated with *KRAS* mutations, but not MSI. Consistent with this, Ogino *et al.* [42] reported that CIMP-low (CIMP-L) tumors, which exhibit DNA methylation at a reduced number of CIMP-related loci, are significantly associated with *KRAS* mutations. CIMP-L tumors are also more frequently observed in men than in women. Hinoue *et al*. [43] identified the CIMP-L subgroup by a genome-scale approach and observed a higher frequency of KRAS mutations compared with other subtypes. However, the frequency of *KRAS* mutation was lower in CIMP-L subgroup than in CIMP2 (∼50% vs. 92%). Yagi *et al.* [44] identified an intermediate-methylation epigenotype (IME) that was associated with *KRAS* mutations. They also demonstrated that CRCs with IME and *KRAS* mutation were correlated with poor prognosis. Although it remains unclear whether these classifications represent the same subgroups of CRCs, these subtypes appear to share overlapping characteristics.

Global DNA hypomethylation may play an important role in genomic instability and colorectal carcinogenesis [45]. Several studies have shown that assessing LINE-1 methylation by quantitative pyrosequencing is highly reproducible, and the levels are correlated with global DNA methylation levels [40], [46]. Here, we revealed that DNs are characterized by LINE-1 hypomethylation and *TP53* mutations. Consistent with these findings, Mastuzaki, *et al*. [47] showed that the LOH at 5q, 8p, or 17p was correlated with a lower level of LINE-1 methylation in CRCs. Furthermore, Ogino *et al*. [40] reported a significant association between LOH at 18q and a low level of LINE-1 methylation in non-MSI-H CRCs and found that LINE-1 hypomethylation was independently associated with shorter survival times of CRC patients. In addition, *TP53* mutations appear to be an important prognostic factor in patients with CRCs [48]. These findings suggest that the relationship between LINE-1 hypomethylation and *TP53* mutation of DNs may be associated with their aggressive behavior.

Yamauchi *et al.* [49] showed that the frequencies of CIMP, MSI, and *BRAF* mutation increase gradually along the bowel from the rectum to the ascending colon, suggesting the colorectal continuum. This phenomenon was confirmed by *BRAF* mutation analysis in an additional study by using a large-scale sample size [50]. In the present study, we evaluated the molecular findings of 158 CRNs based on tumor location (Table S6 in File S1). However, the data corresponding to CIMP, *BRAF*, and MSI-H along bowel subsites was not consistent with the results of the above two studies. This may be due to differences in tumor histology (adenomas vs. cancers or the exclusion of SAs) or different sample size between previous and present studies. However, we found that the frequency of *KRAS* mutation tends to be higher in cecal tumors than any other (75% vs. 14–37%), which is consist with the results of Yamauchi *et al*. [49]. In addition, 5 out of 6 cecal tumors with KRAS mutation were LST-Gs. These findings might be associated with site-specific tumorigenesis.

**Figure 1:**
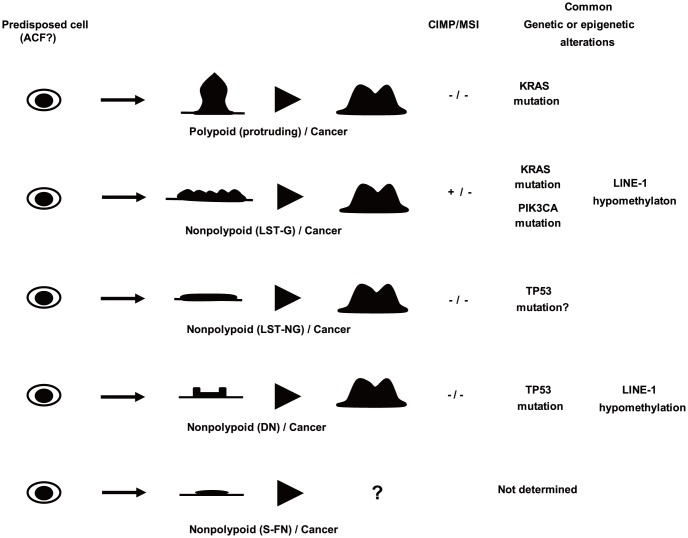
Macroscopic subtypes in colorectal tumorigenesis. Precursor lesions can progress to cancer through the acquisition of epigenetic or genetic changes. Tumors from each subtype exhibit different characteristics, including their underlying molecular and genetic defects. However, whether small flat-elevated neoplasms can progress to other subtypes of CRNs remains unknown. PN, Polypoid neoplasm; LST-G, granular type laterally spreading tumor; LST-NG, non-granular type laterally spreading tumor; S-FN, small flat-elevated neoplasm; DN, depressed neoplasm.

We have proposed the involvement of multiple parallel pathways in colorectal tumorigenesis (Figure 1) [26], [51]. Briefly, age-related methylation such as *SFRP1* alters the physiology of colon stem cells, and predisposes them to acquire additional alterations. Subsequently, predisposed cells follow different pathways on the basis of CIMP and MSI status, and genetic alterations such as *BRAF*, *KRAS*, and *TP53* mutations. We observed increased methylation of *SFRP1* in all subtypes. However, our data revealed that LST-NGs and S-FNs showed no significant genetic or epigenetic changes. There are two possible explanations for the lack of characteristic molecular changes in these tumors. One possibility is that given that most CRCs develop through the adenoma-carcinoma sequence, some tumors could acquire alterations in cancer-related genes in the later stages of colorectal carcinogenesis. Yamamoto *et al*. [52] suggested that DNA copy number aberrations in CRNs occur as a late event in colorectal tumorigenesis. In addition, epigenetic alterations such as the promoter methylation of cancer-related genes begin to occur in precursor lesions such as adenomas. However, most promoter methylation events are more likely to occur during the transition from adenoma to carcinoma [53]. Another possibility is that tumors are more likely to involve other epigenetic alterations such as histone modifications [51] and microRNA changes [54], or the DNA methylation of other critical genes that may be involved earlier in the process of colorectal carcinogenesis. Additional novel molecular changes should therefore be investigated in LST-NG and S-FN cases.

Several studies have demonstrated the potential of molecular biomarkers for the diagnosis or treatment of CRCs. Aspirin could suppress cancer cell growth and induce apoptosis by blocking the PI3K pathway. Importantly, regular use of aspirin after diagnosis was associated with longer survival time in CRC patients with *PIK3CA* mutation, irrespective of aspirin use before diagnosis [55]. We observed a very low frequency of *PIK3CA* mutations (3%) in our tumor samples (adenomas and T1 cancers), consistent with previous studies [37], [56]. However, we observed an increased frequency of *PIK3CA* mutation in LST-G, compared with other types of CRNs, and four of these five lesions histologically revealed HGD or T1 cancer. Kaji *et al.* reported that *PIK3CA* mutations were detected only in LSTs with a higher pathological grade (Cancer or HGD) [56]. In addition, *PIK3CA* mutations are detected in 10–20% CRCs [28], [57], [58]. These observations support the hypothesis that LST-Gs may be premalignant CRC lesions with *PIK3CA* mutations and are associated with the *PIK3CA* mutation being a late observation in colorectal carcinogenesis. The regular use of aspirin may therefore suppress the transition from adenoma to carcinoma.

In summary, we showed that CRNs could be divided into 5 macroscopically distinct subtypes that differ in their DNA methylation status and genetic alterations, suggesting that different mechanisms are involved in colorectal tumorigenesis. However, further studies are required to clarify the epidemiology and clinical progression of the 5 CRN subtypes, which may have implications for the selection of optimal screening programs or therapies.

## Supporting Information

Figure S1 Endoscopic appearance of colorectal neoplasms (all lesions were observed after spraying with indigo carmine dye). (a) Polypoid neoplasm (0-I). (b) Small flat-elevated neoplasm (0-IIa). (c) Depressed neoplasm (0-IIc). (d) Granular type laterally spreading tumor (LST). (e) Non-granular type LST.
(DOC)

File S1 **Contains the files: Table S1.** Summary of the macroscopic classification. **Table S2.** Clinicopathological and molecular features of small flat-elevated neoplasms. S-FN, small flat-elevated neoplasm; proximal, cecum, ascending and transverse colon; distal, descending and sigmoid colon, and rectum; LGD, low grade dysplasia; HGD, high grade dysplasia; MSI-H, high frequency microsatellite instability; CIMP, CpG island methylator phenotype; Mut+, presence of mutation; Mut-, absence of mutation. **Table S3.** The details of the multivariate logistic regressions. a) KRAS mutation: Logistic regression analysis using the stepwise method was performed to evaluate the relationship between KRAS mutation and gender (male vs. female), age (yrs), tumor location (proximal vs. distal), tumor size (mm), macroscopic type (polypoid neoplasm and granular type laterally spreading tumor vs. other types), and histology (T1 cancer vs. adenoma). SD, standard deviation; DF, degree of freedom. b) TP53 mutation: Logistic regression analysis using the stepwise method was performed to evaluate the relationship between *TP53* mutation and gender (male vs. female), age (yrs), tumor location (proximal vs. distal), tumor size (mm), macroscopic type (non-granular type laterally spreading tumor and depressed neoplasm vs. other types), and histology (T1 cancer vs. adenoma). c) PIK3CA mutation: Logistic regression analysis using the stepwise method was performed to evaluate the relationship between PIK3CA mutation and gender (male vs. female), age (yrs), tumor location (proximal vs. distal), tumor size (mm), macroscopic type (granular type laterally spreading tumor vs. other types), and histology (T1 cancer vs. adenoma). d) CIMP: Logistic regression analysis using the stepwise method was performed to evaluate the relationship between CIMP and gender (male vs. female), age (yrs), tumor location (proximal vs. distal), tumor size (mm), macroscopic type (granular type laterally spreading tumor vs. other types), and histology (T1 cancer vs. adenoma). e) LINE-1 hypomethylation: Logistic regression analysis using the stepwise method was performed to evaluate the relationship between LINE-1 hypomethylation and gender (male vs. female), age (yrs), tumor location (proximal vs. distal), tumor size (mm), macroscopic type (granular type laterally spreading tumor and depressed neoplasm vs. other types), and histology (T1 cancer vs. adenoma). **Table S4.** Evaluation of the multivariate logistic regression analysis. DF, degree of freedom. **Table S5.** Clinicopathological and molecular features of serrated adenomas. These data were previously reported by Yano et al. [41]. Four PN and one LST-NG type SAs showed high-grade dysplasia. PN, polypoid neoplasm; LST-G, granular type laterally spreading tumor; LST-NG, non-granular type LST; proximal, cecum, ascending and transverse colon; distal, descending and sigmoid colon, and rectum; MSI-H, high frequency microsatellite instability; CIMP, CpG island methylator phenotype; SSA/P, sessile serrated adenoma/polyp; TSA, traditional serrated adenoma; MP, mixed polyp; Mut+, presence of mutation; Mut-, absence of mutation. **Table S6**. Molecular characteristics of colorectal neoplasms by tumor location. *, P values were calculated by Chi-square test or Kruskal-Wallis test. MSI-H, high frequency microsatellite instability; CIMP, CpG island methylator phenotype; Mut+, presence of mutation; Mut-, absence of mutation.
(DOC)
