# Supplementary figures and images for: Distinct Molecular Features of Different Macroscopic Subtypes of Colorectal Neoplasms
Source: PLoS One. 2014 Aug 5;9(8):e103822. doi: 10.1371/journal.pone.0103822 (PMC4122357; doi:10.1371/journal.pone.0103822)

Figure S1a


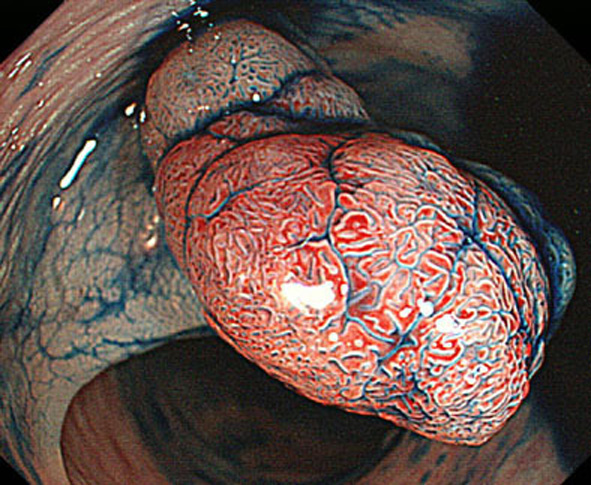


Figure S1b


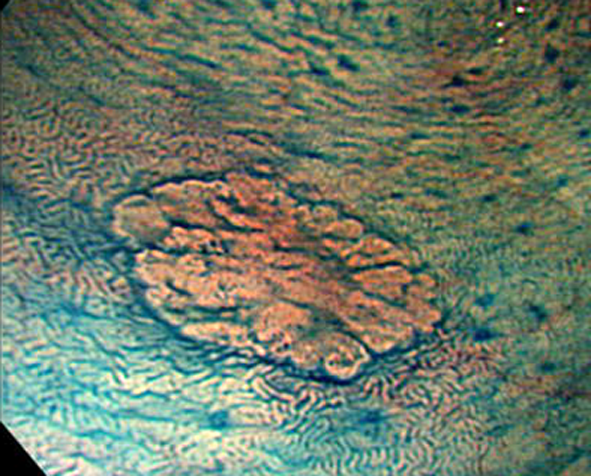


Figure S1c


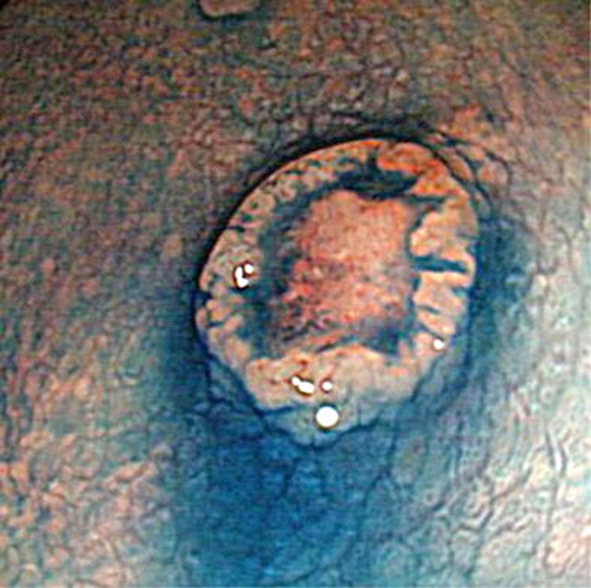


Figure S1d


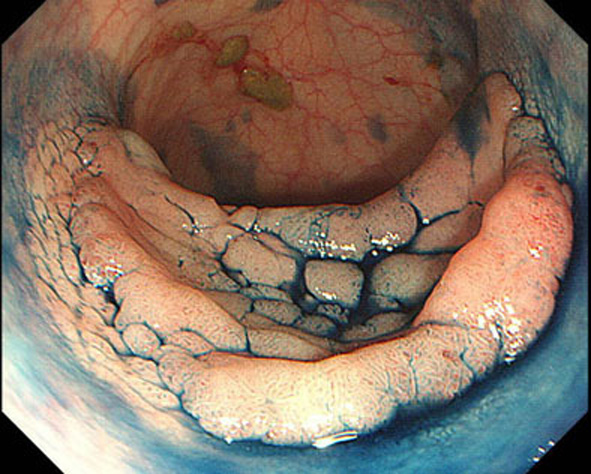


Continued.

Figure S1e


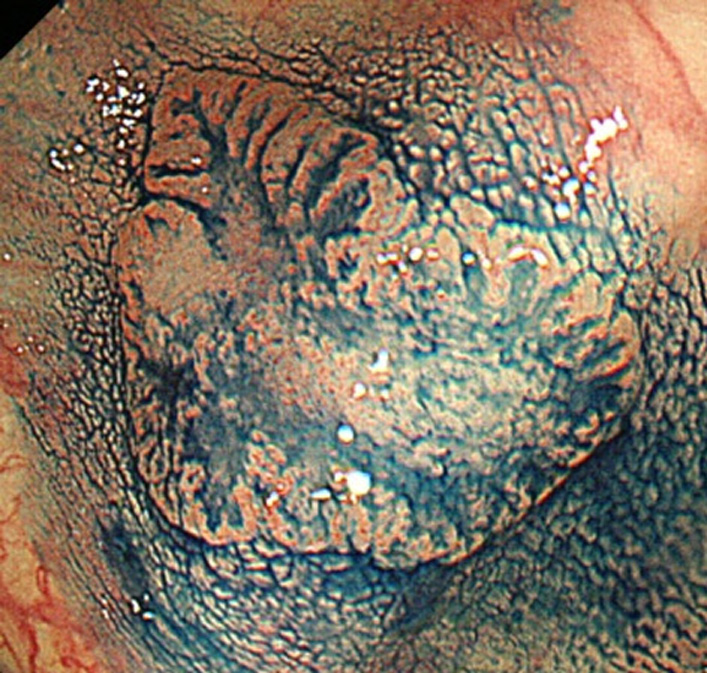

Supplement: Figure S1 — Endoscopic appearance of colorectal neoplasms (all lesions were observed after spraying with indigo carmine dye). (a) Polypoid neoplasm (0-I). (b) Small flat-elevated neoplasm (0-IIa). (c) Depressed neoplasm (0-IIc). (d) Granular type laterally spreading tumor (LST). (e) Non-granular type LST. (DOC) [file pone.0103822.s001.doc]
