# Supplementary material for: Distinct Molecular Features of Different Macroscopic Subtypes of Colorectal Neoplasms
Source: PLoS One. 2014 Aug 5;9(8):e103822. doi: 10.1371/journal.pone.0103822 (PMC4122357; doi:10.1371/journal.pone.0103822)
Supplement: File S1 — Contains the files: Table S1. Summary of the macroscopic classification. Table S2. Clinicopathological and molecular features of small flat-elevated neoplasms. S-FN, small flat-elevated neoplasm; proximal, cecum, ascending and transverse colon; distal, descending and sigmoid colon, and rectum; LGD, low grade dysplasia; HGD, high grade dysplasia; MSI-H, high frequency microsatellite instability; CIMP, CpG island methylator phenotype; Mut+, presence of mutation; Mut-, absence of mutation. Table S3. The details of the multivariate logistic regressions. a) KRAS mutation: Logistic regression analysis using the stepwise method was performed to evaluate the relationship between KRAS mutation and gender (male vs. female), age (yrs), tumor location (proximal vs. distal), tumor size (mm), macroscopic type (polypoid neoplasm and granular type laterally spreading tumor vs. other types), and histology (T1 cancer vs. adenoma). SD, standard deviation; DF, degree of freedom. b) TP53 mutation: Logistic regression analysis using the stepwise method was performed to evaluate the relationship between TP53 mutation and gender (male vs. female), age (yrs), tumor location (proximal vs. distal), tumor size (mm), macroscopic type (non-granular type laterally spreading tumor and depressed neoplasm vs. other types), and histology (T1 cancer vs. adenoma). c) PIK3CA mutation: Logistic regression analysis using the stepwise method was performed to evaluate the relationship between PIK3CA mutation and gender (male vs. female), age (yrs), tumor location (proximal vs. distal), tumor size (mm), macroscopic type (granular type laterally spreading tumor vs. other types), and histology (T1 cancer vs. adenoma). d) CIMP: Logistic regression analysis using the stepwise method was performed to evaluate the relationship between CIMP and gender (male vs. female), age (yrs), tumor location (proximal vs. distal), tumor size (mm), macroscopic type (granular type laterally spreading tumor vs. other types), and h [file pone.0103822.s002.doc]

Table S1

| Macroscopic type |  |
| --- | --- |
| Polypoid neoplasm | Sessile, pedunculated or semipedunculated |
| Nonpolypoid neoplasm | Depressed |
|  | Small flat-elevated |
|  | Granular-type laterally spreading tumor |
|  | Non-granular-type laterally spreading tumor |

Table S2

|  |  | S-FNs (%) |
| --- | --- | --- |
|  |  | (N = 10) |
| Gender | Male | 8 (80) |
|  | Female | 2 (20) |
| Age | Mean, yrs | 66.2 |
|  | (range, yrs) | 42-80 |
| Location | Proximal | 6 (60) |
|  | Distal | 4 (40) |
| Size | Mean, mm | 5.2 |
|  | (range, mm) | 4-8 |
| Histology | LGD | 8 (80) |
|  | HGD+T1 cancer | 2 (20) |
| KRAS | Mut+ | 0 |
|  | Mut- | 10 (100) |
| BRAF | Mut+ | 0 |
|  | Mut- | 10 (100) |
| TP53 | Mut+ | 0 |
|  | Mut- | 10 (100) |
| MSI-H | Presence | 0 |
|  | Absence | 10 (100) |
| CIMP | Presence | 0 |
|  | Absence | 10 (100) |
| MGMT | Mean | 3.1 |
| methylation | 95% CI | 1.0 - 5.2 |
| SFRP1 | Mean | 46.8 |
| methylation | 95% CI | 34.6 – 59.0 |
| RASSF1 | Mean | 3.8 |
| methylation | 95% CI | 1.3 - 6.3 |
| LINE-1 | Mean | 64.0 |
| methylation | 95% CI | 60.9 - 67.1 |

Table S3

1. KRAS mutation

|  |  | β | SD | Wald | DF | P value | Odds ratio (95% CI) |
| --- | --- | --- | --- | --- | --- | --- | --- |
| Step1 | Gender | 0.405 | 0.441 | 0.844 | 1 | 0.358 | 1.499 (0.632-3.559) |
|  | Age | 0.009 | 0.021 | 0.178 | 1 | 0.673 | 1.009 (0.968-1.051) |
|  | Location | 0.733 | 0.445 | 2.716 | 1 | 0.099 | 2.080 (0.870-4.972) |
|  | Macroscopy | 2.377 | 0.572 | 20.370 | 1 | 0.000 | 10.775 (3.838-30.253) |
|  | Size | 0.063 | 0.025 | 6.666 | 1 | 0.010 | 1.065 (1.015-1.118) |
|  | Histology | 0.329 | 0.479 | 0.472 | 1 | 0.492 | 1.390 (0.543-3.557) |
|  | Constant | -5.074 | 1.650 | 9.458 | 1 | 0.002 | 0.006 |
| Step2 | Gender | 0.421 | 0.439 | 0.920 | 1 | 0.338 | 1.524 (0.644-3.602) |
|  | Location | 0.739 | 0.444 | 2.776 | 1 | 0.096 | 2.094 (0.878-4.994) |
|  | Macroscopy | 2.372 | 0.525 | 20.373 | 1 | 0.000 | 10.714 (3.826-30.006) |
|  | Size | 0.062 | 0.024 | 6.549 | 1 | 0.010 | 1.064 (1.015-1.117) |
|  | Histology | 0.336 | 0.478 | 0.492 | 1 | 0.483 | 1.399 (0.548-3.572) |
|  | Constant | -4.463 | 0.763 | 34.182 | 1 | 0.000 | 0.012 |
| Step3 | Gender | 0.422 | 0.439 | 0.925 | 1 | 0.336 | 1.526 (0.645-3.608) |
|  | Location | 0.705 | 0.439 | 2.579 | 1 | 0.108 | 2.024 (0.856-4.788) |
|  | Macroscopy | 2.291 | 0.507 | 20.385 | 1 | 0.000 | 9.881 (3.656-26.707) |
|  | Size | 0.069 | 0.023 | 9.391 | 1 | 0.002 | 1.072 (1.025-1.120) |
|  | Constant | -4.354 | 0.739 | 34.673 | 1 | 0.000 | 0.013 |
| Step4 | Location | 0.646 | 0.432 | 2.232 | 1 | 0.135 | 1.908 (0.817-4.453) |
|  | Macroscopy | 2.318 | 0.507 | 20.879 | 1 | 0.000 | 10.153 (3.757-27.437) |
|  | Size | 0.072 | 0.022 | 10.438 | 1 | 0.001 | 1.075 (1.0029-1.123) |
|  | Constant | -4.229 | 0.721 | 34.400 | 1 | 0.000 | 0.015 |
| Step5 | Macroscopy | 2.209 | 0.494 | 20.013 | 1 | 0.000 | 9.105 (3.459-23.965) |
|  | Size | 0.070 | 0.022 | 10.352 | 1 | 0.001 | 1.073 (1.028-1.120) |
|  | Constant | -3.806 | 0.630 | 36.468 | 1 | 0.000 | 0.022 |

1. TP53 mutation

|  |  | β | SD | Wald | DF | P value | Odds ratio (95% CI) |
| --- | --- | --- | --- | --- | --- | --- | --- |
| Step1 | Gender | -0.728 | 0.661 | 1.215 | 1 | 0.270 | 0.483 (0.132-1.762) |
|  | Age | 0.014 | 0.032 | 0.202 | 1 | 0.653 | 1.015 (0.952-1.081) |
|  | Location | -1.040 | 0.649 | 2.570 | 1 | 0.109 | 0.353 (0.099-1.261) |
|  | Macroscopy | 1.792 | 0.705 | 6.467 | 1 | 0.011 | 6.004 (1.508-23.902) |
|  | Size | -0.025 | 0.038 | 0.441 | 1 | 0.507 | 0.975 (0.905-1.051) |
|  | Histology | 1.583 | 0.728 | 4.728 | 1 | 0.030 | 4.871 (1.169-20.295) |
|  | Constant | -4.220 | 2.448 | 2.972 | 1 | 0.085 | 0.015 |
| Step2 | Gender | -0.711 | 0.658 | 1.167 | 1 | 0.280 | 0.491 (0.135-1.784) |
|  | Location | -0.974 | 0.626 | 2.418 | 1 | 0.120 | 0.378 (0.111-1.289) |
|  | Macroscopy | 1.764 | 0.698 | 6.389 | 1 | 0.011 | 5.836 (1.486-22.914) |
|  | Size | -0.027 | 0.038 | 0.520 | 1 | 0.471 | 0.973 (0.903-1.048) |
|  | Histology | 1.603 | 0.729 | 4.837 | 1 | 0.028 | 4.967 (1.191-20.719) |
|  | Constant | -3.218 | 0.964 | 11.133 | 1 | 0.001 | 0.040 |
| Step3 | Gender | -0.743 | 0.654 | 1.290 | 1 | 0.265 | 0.476 (0.132-1.715) |
|  | Location | -1.002 | 0.625 | 2.569 | 1 | 0.109 | 0.367 (0.108-1.250) |
|  | Macroscopy | 1.807 | 0.695 | 6.752 | 1 | 0.009 | 6.091 (1.559-23.803) |
|  | Histology | 1.439 | 0.691 | 4.337 | 1 | 0.037 | 4.218 (1.088-16.343) |
|  | Constant | -3.634 | 0.810 | 20.144 | 1 | 0.000 | 0.026 |
| Step4 | Location | -0.888 | 0.611 | 2.114 | 1 | 0.146 | 0.411 (0.124-1.362) |
|  | Macroscopy | 1.800 | 0.688 | 6.844 | 1 | 0.009 | 6.049 (1.571-23.301) |
|  | Histology | 1.383 | 0.688 | 4.048 | 1 | 0.044 | 3.988 (1.036-15.348) |
|  | Constant | -3.878 | 0.788 | 24.207 | 1 | 0.000 | 0.021 |
| Step5 | Macroscopy | 1.668 | 0.677 | 6.059 | 1 | 0.014 | 5.299 (1.405-19.992) |
|  | Histology | 1.329 | 0.681 | 3.807 | 1 | 0.051 | 3.779 (0.994-14.364) |
|  | Constant | -4.115 | 0.763 | 29.114 | 1 | 0.000 | 0.016 |

1. PIK3CA mutation

|  |  | β | SD | Wald | DF | P value | Odds ratio (95% CI) |
| --- | --- | --- | --- | --- | --- | --- | --- |
| Step1 | Gender | 0.871 | 1.055 | 0.681 | 1 | 0.409 | 2.389 (0.302-18.894) |
|  | Age | -0.032 | 0.053 | 0.359 | 1 | 0.549 | 0.969 (0.872-1.075) |
|  | Location | 0.445 | 1.075 | 0.171 | 1 | 0.679 | 1.560 (0.190-12.824) |
|  | Macroscopy | 3.276 | 1.348 | 5.907 | 1 | 0.015 | 26.481 (1.855-371.950) |
|  | Size | 0.020 | 0.037 | 0.280 | 1 | 0.597 | 1.020 (0.948-1.097) |
|  | Histology | 0.455 | 1.117 | 0.166 | 1 | 0.684 | 1.576 (0.177-14.064) |
|  | Constant | -3.984 | 3.510 | 1.288 | 1 | 0.256 | 0.019 |
| Step2 | Gender | 0.813 | 1.056 | 0.593 | 1 | 0.441 | 2.255 (0.285-17.870) |
|  | Age | -0.029 | 0.053 | 0.298 | 1 | 0.585 | 0.972 (0.876-1.077) |
|  | Location | 0.318 | 1.036 | 0.094 | 1 | 0.759 | 1.374 (0.180-10.480) |
|  | Macroscopy | 3.092 | 1.251 | 6.106 | 1 | 0.013 | 22.025 (1.895-255.928) |
|  | Size | 0.022 | 0.037 | 0.366 | 1 | 0.545 | 1.022 (0.952-1.098) |
|  | Constant | -3.856 | 3.489 | 1.221 | 1 | 0.269 | 0.021 |
| Step3 | Gender | 0.754 | 1.032 | 0.534 | 1 | 0.465 | 2.125 (0.281-16.052) |
|  | Age | -0.028 | 0.052 | 0.291 | 1 | 0.589 | 0.972 (0.877-1.077) |
|  | Macroscopy | 3.100 | 1.253 | 6.121 | 1 | 0.013 | 22.208 (1.905-258.930) |
|  | Size | 0.025 | 0.036 | 0.472 | 1 | 0.492 | 1.025 (0.955-1.099) |
|  | Constant | -3.760 | 3.450 | 1.188 | 1 | 0.276 | 0.023 |
| Step4 | Gender | 0.633 | 1.000 | 0.401 | 1 | 0.527 | 1.884 (0.265-13.378) |
|  | Macroscopy | 2.943 | 1.217 | 5.843 | 1 | 0.016 | 18.971 (1.745-206.232) |
|  | Size | 0.024 | 0.036 | 0.440 | 1 | 0.507 | 1.024 (0.954-1.099) |
|  | Constant | -5.536 | 1.286 | 18.536 | 1 | 0.000 | 0.004 |
| Step5 | Macroscopy | 2.970 | 1.221 | 5.918 | 1 | 0.015 | 19.499 (1.781-213.474) |
|  | Size | 0.027 | 0.036 | 0.579 | 1 | 0.447 | 1.028 (0.958-1.103) |
|  | Constant | -5.329 | 1.216 | 19.203 | 1 | 0.000 | 0.005 |
| Step6 | Macroscopy | 3.278 | 1.145 | 8.200 | 1 | 0.004 | 26.526 (2.813-250.111) |
|  | Constant | -4.836 | 1.004 | 23.205 | 1 | 0.000 | 0.008 |

1. CIMP

|  |  | β | SD | Wald | DF | P value | Odds ratio (95% CI) |
| --- | --- | --- | --- | --- | --- | --- | --- |
| Step1 | Gender | 0.757 | 0.633 | 1.433 | 1 | 0.231 | 2.132 (0.617-7.366) |
|  | Age | 0.131 | 0.041 | 10.018 | 1 | 0.002 | 1.140 (1.051-1.236) |
|  | Location | -0.034 | 0.641 | 0.003 | 1 | 0.958 | 0.966 (0.275-3.392) |
|  | Macroscopy | 0.353 | 0.758 | 0.203 | 1 | 0.653 | 1.424 (0.306-6.628) |
|  | Size | 0.106 | 0.041 | 6.635 | 1 | 0.010 | 1.111 (1.026-1.204) |
|  | Histology | -0.441 | 0.751 | 0.380 | 1 | 0.538 | 0.643 (0.158-2.615) |
|  | Constant | -14.021 | 3.359 | 17.427 | 1 | 0.000 | 0.000 |
| Step2 | Gender | 0.763 | 0.623 | 1.501 | 1 | 0.221 | 2.145 (0.633-7.270) |
|  | Age | 0.130 | 0.040 | 10.362 | 1 | 0.001 | 1.139 (1.052-1.233) |
|  | Macroscopy | 0.355 | 0.784 | 0.206 | 1 | 0.650 | 1.427 (0.307-6.629) |
|  | Size | 0.105 | 0.041 | 6.658 | 1 | 0.010 | 1.111 (1.026-1.204) |
|  | Histology | -0.436 | 0.710 | 0.377 | 1 | 0.539 | 0.647 (0.161-2.599) |
|  | Constant | -14.009 | 3.347 | 17.515 | 1 | 0.000 | 0.000 |
| Step3 | Gender | 0.796 | 0.617 | 1.664 | 1 | 0.197 | 2.218 (0.661-7.438) |
|  | Age | 0.133 | 0.040 | 11.230 | 1 | 0.001 | 1.143 (1.057-1.235) |
|  | Size | 0.115 | 0.035 | 10.849 | 1 | 0.001 | 1.122 (1.048-1.202) |
|  | Histology | -0.540 | 0.669 | 0.652 | 1 | 0.419 | 0.583 (0.157-2.162) |
|  | Constant | -14.325 | 3.281 | 19.059 | 1 | 0.000 | 0.000 |
| Step4 | Gender | 0.774 | 0.621 | 1.599 | 1 | 0.206 | 2.167 (0.653-7.189) |
|  | Age | 0.132 | 0.039 | 11.231 | 1 | 0.001 | 1.142 (1.056-1.233) |
|  | Size | 0.106 | 0.032 | 10.941 | 1 | 0.001 | 1.112 (1.044-1.184) |
|  | Constant | -14.344 | 3.260 | 19.358 | 1 | 0.000 | 0.000 |
| Step5 | Age | 0.129 | 0.038 | 11.422 | 1 | 0.001 | 1.138 (1.056-1.226) |
|  | Size | 0.110 | 0.032 | 11.986 | 1 | 0.001 | 1.116 (1.049-1.188) |
|  | Constant | -13.844 | 3.129 | 19.575 | 1 | 0.000 | 0.000 |

1. LINE-1 hypomethylation

|  |  | β | SD | Wald | DF | P value | Odds ratio (95% CI) |
| --- | --- | --- | --- | --- | --- | --- | --- |
| Step1 | Gender | 0.049 | 0.416 | 0.014 | 1 | 0.906 | 1.051 (0.465-2.374) |
|  | Age | -0.014 | 0.020 | 0.479 | 1 | 0.489 | 0.986 (0.947-1.026) |
|  | Location | -0.568 | 0.410 | 1.916 | 1 | 0.166 | 0.567 (0.253-1.267) |
|  | Macroscopy | 1.202 | 0.433 | 7.698 | 1 | 0.006 | 3.327 (1.423-7.776) |
|  | Size | 0.019 | 0.020 | 0.873 | 1 | 0.350 | 1.019 (0.980-1.060) |
|  | Histology | 1.386 | 0.432 | 10.303 | 1 | 0.001 | 3.999 (1.716-9.322) |
|  | Constant | -1.408 | 1.451 | 0.941 | 1 | 0.332 | 0.245 |
| Step2 | Age | -0.014 | 0.020 | 0.469 | 1 | 0.494 | 0.968 (0.948-1.026) |
|  | Location | -0.574 | 0.408 | 1.979 | 1 | 0.159 | 0.563 (0.253-1.253) |
|  | Macroscopy | 1.203 | 0.433 | 7.722 | 1 | 0.005 | 3.332 (1.426-7.786) |
|  | Size | 0.019 | 0.020 | 0.934 | 1 | 0.334 | 1.019 (0.980-1.060) |
|  | Histology | 1.385 | 0.432 | 10.290 | 1 | 0.001 | 3.995 (1.714-9.311) |
|  | Constant | -1.407 | 1.451 | 0.940 | 1 | 0.332 | 0.245 |
| Step3 | Location | -0.591 | 0.406 | 2.117 | 1 | 0.146 | 0.554 (0.250-1.228) |
|  | Macroscopy | 1.153 | 0.425 | 7.371 | 1 | 0.007 | 3.166 (1.378-7.276) |
|  | Size | 0.020 | 0.020 | 1.039 | 1 | 0.308 | 1.020 (0.982-1.061) |
|  | Histology | 1.373 | 0.431 | 10.160 | 1 | 0.001 | 3.947 (1.697-9.180) |
|  | Constant | -2.341 | 0.525 | 19.862 | 1 | 0.000 | 0.096 |
| Step4 | Location | -0.563 | 0.404 | 1.945 | 1 | 0.163 | 0.569 (0.258-1.257) |
|  | Macroscopy | 1.253 | 0.414 | 9.165 | 1 | 0.002 | 3.500 (1.555-7.874) |
|  | Histology | 1.453 | 0.423 | 11.771 | 1 | 0.001 | 4.274 (1.864-9.800) |
|  | Constant | -2.022 | 0.409 | 24.471 | 1 | 0.000 | 0.132 |
| Step5 | Macroscopy | 1.227 | 0.407 | 9.070 | 1 | 0.003 | 3.410 (1.535-7.576) |
|  | Histology | 1.481 | 0.421 | 12.365 | 1 | 0.000 | 4.398 (1.926-10.043) |
|  | Constant | -2.289 | 0.375 | 37.178 | 1 | 0.000 | 0.101 |

Table S4

|  | Hosmer-Lemeshow test | | | Accuracy (%) |
| --- | --- | --- | --- | --- |
|  | Chi-square | DF | P value |  |
| KRAS mutation |  |  |  |  |
| Step1 | 8.175 | 8 | 0.417 | 78.3 |
| Step2 | 13.799 | 8 | 0.087 | 77.7 |
| Step3 | 13.563 | 8 | 0.094 | 79.6 |
| Step4 | 0.443 | 6 | 0.998 | 79.6 |
| Step5 | 0.008 | 2 | 0.996 | 79.6 |
| TP53 mutation |  |  |  |  |
| Step1 | 3.101 | 8 | 0.928 | 89.8 |
| Step2 | 6.714 | 8 | 0.568 | 90.4 |
| Step3 | 7.090 | 8 | 0.527 | 90.4 |
| Step4 | 3.704 | 6 | 0.717 | 90.4 |
| Step5 | 1.493 | 2 | 0.474 | 90.4 |
| PIK3CA mutation |  |  |  |  |
| Step1 | 3.311 | 8 | 0.913 | 96.7 |
| Step2 | 5.606 | 8 | 0.691 | 96.7 |
| Step3 | 5.549 | 8 | 0.698 | 96.7 |
| Step4 | 5.425 | 8 | 0.711 | 96.7 |
| Step5 | 1.281 | 2 | 0.527 | 96.7 |
| CIMP |  |  |  |  |
| Step1 | 1.652 | 8 | 0.990 | 92.4 |
| Step2 | 4.205 | 8 | 0.838 | 92.4 |
| Step3 | 3.036 | 8 | 0.932 | 92.4 |
| Step4 | 6.588 | 8 | 0.582 | 92.4 |
| Step5 | 3.655 | 7 | 0.819 | 93.0 |
| LINE-1 hypomethylation |  |  |  |  |
| Step1 | 5.715 | 8 | 0.679 | 74.5 |
| Step2 | 11.068 | 8 | 0.198 | 74.5 |
| Step3 | 15.809 | 8 | 0.045 | 75.2 |
| Step4 | 13.512 | 8 | 0.095 | 74.5 |
| Step5 | 0.033 | 2 | 0.984 | 74.5 |

Table S5

|  |  | PNs  (%) | LST-Gs  (%) | LST-NGs (%) | S-FNs  (%) |
| --- | --- | --- | --- | --- | --- |
|  |  | (N = 68) | (N = 5) | (N = 10) | (N = 3) |
| Gender | Male | 36 (62) | 4 (100) | 7 (78) | 3 (100) |
|  | Female | 22 (48) | 0 | 2 (22) | 0 |
| Age | mean, yrs | 57.5 | 68.8 | 62.3 | 59.7 |
|  | (range, yrs) | (23-77) | (54-85) | (36-82) | (57-61) |
| Location | Proximal | 19 (28) | 5 (100) | 7 (70) | 3 (100) |
|  | Distal | 49 (72) | 0 | 3 (30) | 0 |
| Size | mean, mm | 10.0 | 19.8 | 17.3 | 7.3 |
|  | (range, mm) | (4-25) | (10-35) | (10-30) | (6-9) |
| Histology | SSA/P | 7 (10) | 0 | 7 (70) | 1 (33) |
|  | TSA | 50 (74) | 2 (40) | 2 (20) | 2 (66) |
|  | MP | 11 (16) | 3 (60) | 1 (10) | 0 |
| *KRAS* | Mut + | 11 (16) | 0 | 2 (20) | 0 |
|  | Mut - | 57 (84) | 5 (100) | 8 (80) | 3 (100) |
| *BRAF* | Mut + | 40 (59) | 2 (40) | 3 (30) | 2 (66) |
|  | Mut - | 28 (41) | 3 (60) | 7 (70) | 1 (33) |
| MSI-H | presence | 4 (6) | 0 | 0 | 0 |
|  | absence | 64 (94) | 5 (100) | 10 (100) | 3 (100) |
| CIMP | presence | 22 (32) | 4 (80) | 6 (60) | 1 (33) |
|  | absence | 46 (68) | 1 (20) | 4 (40) | 2 (66) |

Table S6

| Molecular  features |  | Total | Cecum | Ascending | Transverse | Descending | Sigmoid | Rectum | *P* value |
| --- | --- | --- | --- | --- | --- | --- | --- | --- | --- |
| *KRAS* | Mut+ | 43 (27%) | 9 (75%) | 2 (14%) | 12 (24%) | 3 (21%) | 10 (20%) | 7 (37%) | 0.0036 |
|  | Mut- | 115 (73%) | 3 (25%) | 12 (86%) | 38 (76%) | 11 (79%) | 39 (80%) | 12 (63%) |  |
| *BRAF* | Mut+ | 3 (2%) | 0 (0%) | 0 (0%) | 1 (2%) | 1 (7%) | 1 (2%) | 0 (0%) | 0.7083 |
|  | Mut- | 155 (98%) | 12 (100%) | 14 (100%) | 49 (98%) | 13 (93%) | 48 (98%) | 19 (100%) |  |
| *TP53* | Mut+ | 15 (9%) | 0 (0%) | 1 (7%) | 4 (8%) | 0 (0%) | 5 (10%) | 5 (26%) | 0.1001 |
|  | Mut- | 143 (91%) | 12 (100%) | 13 (93%) | 46 (92%) | 14 (100%) | 44 (90%) | 14 (74%) |  |
| *PIK3CA* | Mut+ | 5 (3%) | 3 (25%) | 0 (0%) | 0 (0%) | 0 (0%) | 2 (4%) | 0 (0%) | 0.0005 |
|  | Mut- | 146 (97%) | 9 (75%) | 14 (100%) | 50 (100%) | 14 (100%) | 47 (96%) | 19 (100%) |  |
| MSI-H | Presence | 5 (3%) | 0 (0%) | 2 (14%) | 2 (4%) | 0 (0%) | 1 (2%) | 0 (0%) | 0.1901 |
|  | Absence | 153 (97%) | 12 (100%) | 12 (86%) | 48 (96%) | 14 (100%) | 48 (98%) | 19 (100%) |  |
| CIMP | Presence | 17 (11%) | 3 (25%) | 1 (7%) | 5 (10%) | 0 (0%) | 3 (6%) | 5 (26%) | 0.0664 |
|  | Absence | 141 (89%) | 9 (75%) | 13 (93%) | 45 (90%) | 14 (100%) | 46 (94%) | 14 (74%) |  |
| LINE-1 methylation | Mean (%) | 61.7 | 59.2 | 62.8 | 62.7 | 63.3 | 61.4 | 59.1 | 0.0384 |
|  | 95%CI | (60.9-62.4) | (57.8-60.5) | (61.6-64) | (61.7-63.8) | (61-65.5) | (60-62.9) | (55.7-62.5) |  |
